# An observational study of associations among maternal fluids during parturition, neonatal output, and breastfed newborn weight loss

**DOI:** 10.1186/1746-4358-6-9

**Published:** 2011-08-15

**Authors:** Joy Noel-Weiss, A Kirsten Woodend, Wendy E Peterson, William Gibb, Dianne L Groll

**Affiliations:** 1 School of Nursing, University of Ottawa, 451 Smyth Road, Ottawa, ON, K1H 8M5, Canada; 2 Trent-Fleming School of Nursing, 1600 West Bank Drive, Peterborough, ON, K9J 7B8, Canada; 3 Departments of Obstetrics and Gynaecology, Cellular and Molecular Medicine, University of Ottawa, 501 Smyth Road, Ottawa, ON, K1H 8L6, Canada; 4 Department of Psychiatry, Queen's University, 752 King Street West, Kingston, ON, K7L 4X3, Canada

## Abstract

**Background:**

Newborn weight measurements are used as a key indicator of breastfeeding adequacy. The purpose of this study was to explore non-feeding factors that might be related to newborn weight loss. The relationship between the intravenous fluids women receive during parturition (the act of giving birth, including time in labour or prior to a caesarean section) and their newborn's weight loss during the first 72 hours postpartum was the primary interest.

**Methods:**

In this observational cohort study, we collected data about maternal oral and IV fluids during labour or before a caesarean section. Participants (n = 109) weighed their newborns every 12 hours for the first three days then daily to Day 14, and they weighed neonatal output (voids and stools) for three days.

**Results:**

At 60 hours (nadir), mean newborn weight loss was 6.57% (SD 2.51; n = 96, range 1.83-13.06%). When groups, based on maternal fluids, were compared (≤1200 mls [n = 21] versus > 1200 [n = 53]), newborns lost 5.51% versus 6.93% (p = 0.03), respectively. For the first 24 hours, bivariate analyses show positive relationships between a) neonatal output and percentage of newborn weight lost (r(96) = 0.493, p < 0.001); and b) maternal IV fluids (final 2 hours) and neonatal output (r(42) = 0.383, p = 0.012). At 72 hours, there was a positive correlation between grams of weight lost and all maternal fluids (r(75) = 0.309, p = 0.007).

**Conclusions:**

Timing and amounts of maternal IV fluids appear correlated to neonatal output and newborn weight loss. Neonates appear to experience diuresis and correct their fluid status in the first 24 hours. We recommend a measurement at 24 hours, instead of birth weight, for baseline when assessing weight change. Because practices can differ between maternity settings, we further suggest that clinicians should collect and analyze data from dyads in their care to determine an optimal baseline measurement.

## Background

A planned caesarean section, fetal decelerations before birth, and an epidural in labour often have something in common: boluses of maternal intravenous fluids administered before birth [1-3]. This extra maternal fluid raises a question about how the unborn neonate is affected and whether the ensuing post-birth newborn weight loss is related to a fluid shift rather than feeding or pathology.

**Figure 1:**
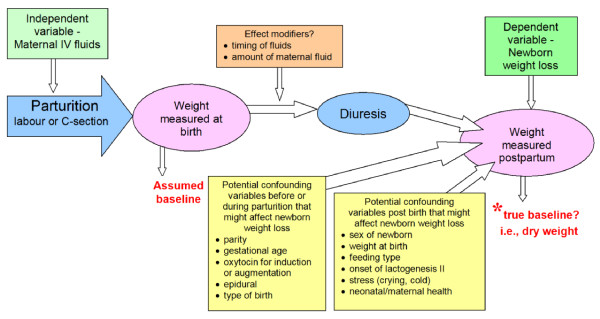
**Conceptual framework includes potential confounding variables and effect modifiers**.

The purpose of this research study was to analyze how maternal fluids in labour or before a caesarean section are related to newborn weight loss during the first three days following birth. Specifically, we hypothesized that there would be a positive relationship between maternal intravenous (IV) fluids received during parturition (the act of giving birth) and 1) weight lost by the newborn and 2) neonatal output (i.e., voids and stools) during the first 72 hours post birth. We also hypothesized there would be a positive relationship between neonatal output and newborn weight loss during the first three days (see Figure 1 for conceptual map of the variables).

### Newborn weight loss

Newborns are typically weighed within a few minutes following birth, and that measurement becomes the baseline for monitoring newborn weight loss. Health care professionals use the percentage of weight change from birth weight as an indicator of feeding adequacy and usually attribute weight loss to inadequate intake as a result of insufficient milk supply or ineffective milk transfer [4-7]. Clinical practice guidelines suggest a weight loss of more than 7% from birth weight is cause for concern [4-7].

Although some patterns for weight loss appear in the literature, there is a lack of evidence to explain the variation in early newborn weight loss and a lack of indicators for morbidity and mortality related to a percentage of weight lost during the first two weeks postpartum [8]. Even so, weight loss patterns are used as the basis for clinical decisions about infant feeding.

Recently, researchers looked at non-feeding factors related to neonatal weight loss [9-12]. Martens and Romphf conducted a chart audit (n = 812) and determined that higher birth weight, female sex, epidural use, and longer hospital stay were positively related to neonatal weight loss in hospital [9]. With some of these variables, causality is ambiguous, for example, the longer hospital stay might be the result of the weight loss not the cause of the loss. Lamp and Macke analyzed data related to maternal intrapartum fluid intake from admission to birth, and neonatal weight, output, and feedings in the first 48 hours [10]. They found the maternal fluids were not related to neonatal weight loss, but the number of diapers was predictive of the weight loss. Mulder et al. also found total voids to be a significant predictor of neonatal weight loss [11]. Most recently, Chantry et al set out to identify modifiable risk factors for excess neonatal weight loss [12]. The variables they studied are generally associated with breastfeeding exclusivity and duration (i.e., maternal age, education, and income), but they found that two variables, hourly intrapartum maternal fluid rates and delayed lactogenesis (> 72 hours), were predictive of weight loss measured at three days postpartum [12].

### Fetal fluid regulation

At the beginning of the fetal period, the fetus is about 95% water, and this percentage decreases to about 70% at birth with the fluid transitioning from extracellular spaces to intracellular spaces closer to birth [13]. Throughout fetal life, the proportion of fluid in the intravascular space remains constant, and the purpose seems to be maintenance of a constant intravascular fluid level and system homeostasis with optimal blood volume [13]. As the fetus grows, it swallows and "breathes" the amniotic fluid and, in doing so, voids and "exhales" into the amniotic fluid [14].

In the short term, the key mechanisms for fetal fluid homeostasis seem to be transcapillary and transplacental fluid movement [13]. As early as 1960, Battaglia et al. demonstrated that pregnant women and their fetuses are inextricably linked via the placenta, and that fluid and electrolytes move freely between the two separate bloodstreams [15]. Fluid balance in the fetus is essentially maintained through mechanisms of diffusion, osmosis, and active transport [16]. Along with the renal and circulatory systems processes for maintaining fluid balance, intramembranous fluid movements have been demonstrated in sheep and may be active in the human fetus [17]. During the transition from fetal life, the newborn's kidneys begin to process about 10% of cardiac output in contrast to the 1.9% of cardiac output that fetal kidneys receive [18].

### Use of intravenous fluids during parturition

To maintain maternal hemodynamics, IV fluids are used as boluses and continuously during parturition [1,2]. There are a few reports that speculate about or demonstrate links between maternal IV fluids given before birth and neonatal weight loss. Kepplar discussed the use of IV fluids in labour and proposed the potential for excess neonatal weight loss [19]. Dahnberg et al. showed that infants of mothers who received IV fluids had hyponatremia and lost 50% more weight than infants whose mothers only received oral fluids (6.17% ± 3.36 SD versus 4.07% ± 2.20 SD, p < 0.01) [20]. Researchers have recently begun to question the effect of maternal fluids during parturition on neonatal weight loss [10,12]. The hypotheses for this study presuppose that women receive IV fluids for medical reasons [1,2], fluids move freely from a woman to her fetus [15], the newborn is overhydrated due to iatrogenic factors [20], and a correction in the newborn's fluid balance is a measurable weight loss.

Optimum infant health requires adequate breastfeeding; inadequate milk intake may create health risks for an infant [21-23]. At the same time, there are risks associated with using infant formula [24-27]. Clinicians (e.g., nurses, lactation consultants, and physicians working with breastfeeding women) need to understand the factors that affect newborn weight loss to: (a) account for weight loss that requires no intervention; (b) prevent unnecessary weight loss; and (c) recommend appropriate interventions when required due to weight loss. In this study, we investigated iatrogenic factors that may require no intervention.

## Methods

This research study was an observational, prospective cohort design with a convenience sample of pregnant women. It followed participants from labour or prior to a caesarean section to two weeks postpartum, and it was designed to collect data about factors that might influence newborn weight loss. The University of Ottawa Research Ethics Board and the research ethics boards of each hospital provided ethics approval based on the requirements of the Tri-Council Policy Statement [28].

### Recruitment

The study was advertised through brochures and posters in the community, at ultrasound clinics, and in physicians' and midwives' offices. The study information was presented at hospital tours and prenatal classes. Active recruitment involved one-to-one explanations with pregnant women and their partners.

Inclusion criteria included: expecting a fullterm (end of 36 6/7 weeks - 259 days) [29], single, healthy infant at one of the participating hospitals or a home birth, and planning to breastfeed. Healthy was defined as both mother and newborn discharged at the same time and able to breastfeed without restriction. All parities were included. Women completed and returned a contact information sheet, consent for the study, consent for a chart audit, and a prenatal questionnaire before their baby's birth.

### Measurements and data collection

The amounts of oral and IV fluid (in millilitres) during labour or prior to a caesarean section were collected from admission to birth. Since most nurses in the study area work 12-hour shifts, fluid data were collected in 12 hour segments. Parents normally recorded oral intake and nurses reported IV fluids. The IV amount was reported independently of regular charting because charted IV fluids are recorded as totals until the intravenous is discontinued which is normally after the birth. For this study, fluid amounts after the cord was cut were not counted as they no longer could affect the fetus. Timing of IV fluids, with the rationale that fluids could resettle and move back from fetus to mother, was studied in a subset of the participants based on data availability. Lamp and Macke's results supported our supposition that timing mattered [10]. In the final months of the study, the IV fluids in the final two hours were also reported by nurses, and we audited the collected data to isolate two-hour pre-birth IV amounts where possible.

Women received a study baby scale when they arrived at the mother-baby unit. Parents took the scales home when discharged, and all weights were measured on the same scale for consistency. In the case of home births, the scale was provided before the expected due date. We used the Ultrascale MBSC-55 Digital Scale with precision to within 2 grams under 500 grams (the diapers) and to within 10 grams over 500 grams (the baby) [30]. The scales ran on batteries. The researcher sanitized each scale and tested it using a standardized weight. If there was a question about its accuracy, it was recalibrated according to manufacturer's directions. Because birth weight was the first weight measured by the nurses on the hospital's scale, the same standardized weight was used with the hospitals' scales to ensure uniformity.

We provided two data collection sheets, one to record diaper weights and one for baby weights, and an instruction sheet with the scale. All babies were weighed without clothing. Parents weighed their newborns every 12 hours for 72 hours, then daily from Days 4 to 14. All weights were recorded in grams or converted if parents used pounds and ounces.

Starting at birth, parents weighed and recorded all diapers (included voids and stools) for three days. If nurses changed a diaper, it was saved in a plastic bag. Each diaper was weighed and the full weight was recorded. Since diapers came from various sources (e.g., provided by hospital, brought from home), the weight of a dry diaper was also recorded, and the researcher subtracted the dry weight before totalling the weights for each 24-hour set of diapers. For one hospital, a neonatal security band was used and parents indicated whether the band was on. The researcher subtracted its weight (i.e., 22 grams) when required.

Infant feeding categories were established by asking mothers if babies were supplemented in hospital, and with the use of an algorithm designed to determine feeding category [31,32]. At 14 days postpartum, the researcher called participants to complete the telephone questionnaire. Arrangements were made to pick up the baby scale and data collection sheets from their homes.

### Analysis

Descriptive statistics and tests of significance were undertaken using SPSS 18. The intention was to describe the participants and to test for correlations between the variables. An a priori sample size calculation determined that correlations analyses (i.e., Pearson's correlation and Spearman's rho) required a sample of 82 subjects to detect a moderate (0.30) correlation at an alpha of 0.05 with a power of 0.80 for a two tailed test [33]. Attrition was expected to be about 25%.

Supplemented babies remained in the study and were not treated differently from exclusively breastfed babies. This decision was based on generalizability and evidence that there is little difference in weight loss in the first days post partum [9,10]. When the percentage weight loss at 60 hours (nadir of weight loss) for the supplemented versus non-supplemented newborns was compared, the mean losses were 6.9% and 6.5%, respectively. Using an independent T-test, there was no statistically significant difference (p > 0.52). No fully formula fed babies stayed in the study.

## Results

### Description of setting and sample

Data were collected from January 2008 to June 2010 in Ontario, Canada at five sites: two small community hospitals (~ 300-400 births per year each), a large community hospital (~ 2500 births per year), and a teaching hospital with two sites (~6800 births combined sites) [34]. One hundred and sixty-four women registered for the study, and 109 families completed data collection. Reasons for loss of registered participants included: 37% who intended to continue but did not receive a baby scale; 23% who stopped because of illness (e.g., infant's prematurity, unexpected caesarean section, postpartum hemorrhage); 17% who changed their mind about the study or opted to formula feed; and for 23% the exact reason is not known. The group that completed the study was comparable to the lost participants based on age, amount of breastfeeding, maternal education level, and family income (using independent T-test and chi-squared, p < 0.05). There was a difference between the two groups based on being in a committed relationship (100% versus 88% respectively, p > 0.05). Demographic characteristics of the participants, mothers and newborns who completed the study, are in Table 1.

**Table 1:** Characteristics of mothers and newborns

Characteristics	Frequency	n
Maternal age (years) [*+/-*SD(range)]	32 *+/- *4.3 (22-45)	106
Committed relationship (%)	108 (100)	108
Completed post-secondary education (%)	88 (81)	108
Family income > 70 K (CAN) (%)	90 (83)	107
Never smoked (%)	93 (86)	108
Primiparous women (%)	46 (42)	108
Multiparous women's years of breastfeeding experience - all children [median (range)]	1 year (0.10-5.30)	108
Decided to breastfeed before pregnant (%)	97 (92)	106
Plan to exclusively breastfeed for 6 months (%)	84 (83)	101
Planned to breastfeed for 1 year or more (%)	78 (76)	102
Gestation (weeks) [*+/-*SD(range)]	39.8 *+/- *1.2 (37-42)	109
Care at birth (%)		
Obstetrician	89 (82)	
Family physician	13 (12)	109
Midwife	7 (6)	
Birth type (%)		
Vaginal	82 (75)	
Planned caesarean	13 (12)	109
Unplanned caesarean	14 (13)	
Intravenous inserted before birth (%)	80 (78)	102
Epidural for vaginal birth (%) n = 82	52 (63)	108
Oxytocin for induction or augmentation (%)	50 (46)	109
Late onset of lactogenesis II [> 72 hours] (%)	43 (41)	104
Newborn sex (%)		
Female	55 (51)	109
Male	54 (49)	
Newborn birth weight (grams) [*+/-*SD(range)]	3619 *+/- *502 (2185-4707)	108
Supplemented in hospital (%)	28 (27)	104
Treated for jaundice (%)	11 (10)	109
Feeding categories at 2 weeks (%)		
Exclusively breastfed (breast milk from birth)	67 (62)	
Totally breastfed (no supplements in Week 2)	15 (14)	108
Predominant breast milk (1-2 supps/day in Week 2)	14 (13)	
Partial or no breast milk (> 2 supps/day in Week 2)	12 (11)	

### Weight loss and maternal fluids

Tables 2 and 3 present the average weight loss from birth (in grams and by percentage) and the amounts of maternal fluid intake during parturition, respectively. Bivariate analyses compared millilitres of maternal intake with grams of newborn weight lost. Oral fluids alone were not significant. Intravenous fluids (both total from admission and two-hour pre-birth amounts) and combined IV and oral fluids were statistically significant at 60 hours (see Table 4).

**Table 2:** Average weight loss from birth (N = 109)

Average weight loss in grams from birth			
***Timing***	***Sample size***	***Mean +/- SD***	***Range***

12 hours	95	84.95 *+/- *46.1	00 to 191
24 hours	97	159.74 *+/- *52.5	55 to 292
36 hours	98	214.80 *+/- *56.3	78 to 357
48 hours	105	236.96 *+/- *72.2	56 to 407
60 hours	96	237.20 *+/- *98.4*	70 to 467
72 hours	100	210.10 *+/- *101.9	(20)** to 437

**Average percentage weight loss from birth**			

*Timing*	*Sample size*	*Mean +/- SD*	*Range*

12 hours	95	2.34 *+/- *1.21	000 to 4.77
24 hours	97	4.45 *+/- *1.41	1.47 to 7.66
36 hours	98	5.94 *+/- *1.45	2.10 to 8.96
48 hours	105	6.55 *+/- *1.82	1.64 to 10.23
60 hours	96	6.57 *+/- *2.51*	1.83 to 13.06
72 hours	100	5.78 *+/- *2.58	(0.53)** to 11.15

* Timing of maximum weight loss

** Numbers in parentheses indicate weight gain

**Table 3:** Amounts of maternal fluids in millilitres (N = 109)

Category of fluids	n	Mean (SD)	Median	Range
IV fluids in last 2 hrs before birth	43	713 (852)	400	0 to 3100
IV fluids admit to birth	93	1578 (1215)	1500	0 to 5800
Oral fluids admit to birth	82	626 (672)	400	0 to 3000
All fluids admit to birth	81	2129 (1516)	1850	0 to 7200

Data were not normally distributed

Shapiro-Wilk test used to determine normality

**Table 4:** Newborn weight loss in grams correlated to maternal fluid types (N = 109)

	Type and timing of maternal fluid		
***Timing of weight loss***	***IV fluids 2 hrs before birth***	***IV fluids admit to birth***	***Oral and IV fluids admit to birth***

Birth to 60 hrs	.406, p = 0.011 n = 38	.216, p = 0.050 n = 83	.275, p = 0.018 n = 74
Birth to 72 hrs	.273, p = 0.092 n = 39	.223, p = 0.039 n = 86	.309, p = 0.007 n = 75

Spearman's rho correlation coefficient, 2-tailed used for all tests

No significant relationship between newborn weight loss and maternal fluids before 60 hours

### Maternal fluids and neonatal output

Details of neonatal output are presented in Table 5. Discrete outputs (i.e., daily total amounts of voids and stools) on Day 1 were positively correlated to two-hour pre-birth IV amounts, but not to other fluid categories. Neither Day 2 nor Day 3 neonatal outputs were correlated to any maternal IV fluids amounts (see Table 6).

**Table 5:** Average neonatal output (N = 109) in grams

*Timing*	*n*	*Mean (SD)*	*Median*	*Range*
0 - 24 hrs	107	83.04 (47.8)	76	0 to 314
24 - 48 hrs	107	84.43 (44.8)	69	14 to 230
48 - 72 hrs	106	133.30 (86.5)	97	22 to 440

Data were not normally distributed

Shapiro-Wilk test used to determine normality

**Table 6:** Maternal fluid amounts correlated to neonatal output (N = 109)

	Category of maternal fluid		
***Neonatal output***	***IV fluids 2 hrs before birth***	***IV fluids admit to birth***	***All fluids admit to birth***

0 to 24 hrs	0.383, p = 0.012 n = 42	0.115, p = 0.276 n = 92	0.080, p = 0.480 n = 81
24 to 48 hrs	0.241, p = 0.124 n = 42	0.171, p = 0.102 n = 92	0.043, p = 0.703 n = 81
48 to 72 hrs	0.110, p = 0.495 n = 41	0.021, p = 0.846 n = 91	-0.054, p = 0.632 n = 80

Spearman's rho correlation coefficient, 2-tailed used for all tests

### Neonatal output and newborn weight loss

Using bivariate analyses, newborn weight loss as a percentage for each 24 hour period was correlated to neonatal output for the same period (see Table 7). For Day 1, there was a positive relationship between neonatal output and newborn weight loss (i.e., as weight of diaper increases, weight loss increases). This result indicates that as neonatal output increases, newborn weight loss also increases during the first 24 hours. On Day 2, there was no relationship between the two variables. On Day 3, there was a statistically significant *negative *correlation between output and weight loss. Since we are looking for weight loss, this final result is essentially a double negative indicating as weight of diaper increases, weight loss decreases (i.e., newborn gains).

**Table 7:** Percentage of newborn weight loss correlated to neonatal output (N = 109)

	Timing of neonatal output		
***Time of weight loss***	***0-24 hours*** ***(n = 96)***	***24-48 hours*** ***(n = 95)***	***48-72 hours*** ***(n = 98)***

Birth to 24 hours	0.493 p < 0.001	--	--
24 to 48 hours	--	-0.107 p = 0.303	--
48 to 72 hours	--	--	-0.351 p < 0.001

Spearman's rho correlation coefficient, 2-tailed used for all tests

### Comparing based on groups

By forming two groups based on total amount of fluids received, we could compare the two groups and look for differences in amounts of newborn weight loss. We used the point of maximum weight loss (60 hours) and chose 1200 mls as the maternal fluid amount to compare. Our rationale was multifold: 1) the 25th percentile of fluids was 1200 mls; 2) this amount represented the median of oral fluids plus half of the IV amounts; and 3) most births were within 12 hours of admission - 100 mls per hour is a reasonable maintenance quantity. For the mothers who had 1200 mls or less, the average percentage of newborn weight loss at 60 hrs was 5.51% (n = 21). Whereas the group with more than 1200 mls total fluids, their babies averaged a 6.93% weight loss (n = 53). The difference of 1.42% was statistically significant (p = 0.03).

### Lactogenesis II

Women were asked if they noticed the day their "milk came in" (birth counted as Day 1), and 95% reported the day they noticed first day of breast fullness. There was a significant positive correlation between late onset of lactogenesis II (> Day 3, reported by 41% of sample) and percentage of newborn weight loss at 72 hours (r_s_(97) = 0.380, p < 0.001, two-tailed Spearman's rho). Additionally, the reported late onset of lactogenesis II was positively related to the total amounts of maternal fluids from admission to birth (r_s_(78) = 0.307, p = 0.006, two-tailed Spearman's rho).

### Regression analysis

In addition to the two independent variables of interest, maternal fluids and neonatal output, several variables were identified in the literature as predictors of weight loss. The variables cited in the literature were used in the conceptual model (see Figure 1): parity [35] gestational age [9], oxytocin [induction or augmentation] use [35], epidural use [36], type of birth [37], infant sex, [9,10] birth weight [9,10], feeding type (i.e., supplemented) [9,10], timing of lactogenesis II [38], and time with skin-to-skin [39].

Bivariate analyses show a positive correlation between weight loss at 60 hours and the two key independent variables, maternal two-hour pre-birth IV fluids (r_s_(38) = 0.383, p = 0.018) and the first day of neonatal output (r_s_(95) = 0.287, p = 0.005, two-tailed Spearman's rho). When these two variables are analyzed together in a regression analyses, the fluid remains significant (p = 0.05), but output is not significant (p = 0.202). Using the variables in this model and holding neonatal output at 94.82 mls, we predict that 250 mls of maternal fluids results in 5.78% weight loss; whereas, 2500 mls of maternal fluids results in 8.03% weight loss.

We also ran a multiple linear regression analysis to determine predictive variables for percentage of weight loss at 60 hours - the nadir of loss. Gestational age, birth weight, and onset of lactogenesis II were included in the model with two-hour pre-birth IV fluids. We eliminated variables when a bivariate analysis resulted in p-values of greater than 0.20. Consequently, parity, oxytocin, infant sex, and supplemented (yes/no) were not included in the model. Epidural (p = 0.157, n = 95) and birth type (p = 0.008, n = 95) met the initial criteria, but both variables were strongly associated with the two-hour pre-birth IV fluids (r > 0.70, p < 0.001) and thus excluded. There was inadequate data to include skin-to-skin in the model.

Overall, the regression model was significant (see Table 8 for details). The two-hour pre-birth maternal fluids remained predictive of *percentage of weight lost *when gestational age, birth weight, and timing of lactogenesis II were included in the model. Gestational age, birth weight, and timing of lactogenesis II were not predictive at 60 hours postpartum. In contrast, at 72 hours, the maternal fluids were not predictive (p = 0.384), but timing of lactogenesis II was predictive (p = 0.019; model not shown). When the dependent variable in the model was changed to *grams of weight lost *at 60 hours, both maternal two-hour pre-birth IV fluids and birth weight were predictors of weight loss (p = 0.022 and p = 0.036, respectively).

**Table 8:** Regression analysis of predictor variables for percentage of neonatal weight loss at 60 hours postpartum (n = 37)

Variable	B	*β*	Sr2* (incremental)	p
Constant	15.507			
2-hr pre-birth IV fluids (mls)	0.001	0.371	0.076	0.024
Gestational age (weeks)	-0.339	-0.143	-	0.376
Birth weight (grams)	0.000	0.071	-	0.657
Onset of lactogenesis II (1 </= 72 hours, 2 > 72 hours)	1.484	0.254	-	0.104

R^2 ^= 0.286 (F_4,33 _= 3.310, p = 0.022) (Adjusted R^2 ^= 0.200)

Unique variability = 0.076, Shared variability 0.210

* *Sr2 *only reported for significant predictors

## Discussion

The hypotheses were, for the most part, supported and there were additional notable findings. There were positive relationships among the variables: maternal IV fluids, neonatal output, and newborn weight loss, although the relationship between maternal IV fluids was not evident until 60 hours postpartum. We are not certain if this result is because it was the nadir, therefore the greatest effect size, or because it took time for fluid and weight to settle.

The neonatal output results were the most intriguing and unexpected. The hypotheses partially held. The positive correlation between maternal IV fluids and neonatal output were limited in both type and timing. Specifically, only the two-hour pre-birth maternal fluid amount was statistically significant for the first 24 hours. The relationship between neonatal output and newborn weight loss indicates each of the three days has a different correlation, as the correlation moves from positive to no relationship to a negative relationship. Only the first 24 hours of neonatal output and newborn weight loss were positively correlated. We interpret these results to suggest the newborn experiences diuresis, but only in the first 24 hours. Our interpretation is substantiated by the finding maternal IV fluid is only related to neonatal output on the first day.

### Maternal fluids

There is little information in the literature about the relationship between IV fluids and neonatal weight loss in the first week postpartum. In a recent study, Lamp and Macke found no relationship between intrapartum maternal fluids and neonatal weight loss [10]. There are three main differences between their study and this study: (a) data were collected for 48 hours versus 72 hours and 14 days, respectively; (b) the amounts of fluids from admission to birth were quite different (2522.5 to 5013.75 mls. versus 0 to 7200 mls. for our study); (c) all fluids in Lamp and Macke's study were measured from admission to birth (i.e., they did not collect data about IV fluids specifically within two hours of birth) [10]. With our study, correlations between fluids and weight loss appear at 60 hours and 72 hours. The wider range of fluid amounts and longer data collection period may account for positive findings in this study. More recently, Chantry et al. found an association between excess weight loss and maternal intrapartum fluid balance [12]. The neonates in their study were weighed at three days, so it appears they captured the timing when the correlation appears.

### Neonatal output

Neonatal output in our study was significantly related to weight change. These findings corroborate results from three studies. Lamp and Macke [10] observed that the number of wet diapers was predictive of weight loss and Mulder et al. [11] reported that total voids were a predictor of excessive weight loss (> 7%). Chantry et al. [12] compared number of neonatal voids in the first four hours with categories of maternal fluids and determined a positive relationship. Their methods differ from our design, as we report the days separately and output as total weight of diapers.

It appears lactogenesis II affects output on Day 3 when the relationship between output and weight loss became negative. A negative relationship indicates one of two possibilities: the neonate who has increased output has increased weight gain or the newborn with decreased output is losing weight.

### Generalizability

Overall, this convenience sample is comparable to Ontario, the provincial population of origin. In hospital, 27% of study participants supplemented their babies, and the provincial rate of hospital supplementation is 28% [34]. In 2007-08, 43% of women who gave birth in Ontario were first-time mothers [34]. Likewise, 42.2% of the study participants are primiparous. The study participants also have similar rates of caesarean sections and epidural use when compared to Ontario provincial rates (25% versus 28.4% and 64% versus 62%, respectively) [34].

Comparisons of neonatal weight loss are difficult because some authors count birth as Day 1 and others treat the first post-birth day as Day 1 (i.e., birth = Day 0), and it is often not clear which was used. The Day 1 weight for this study is birth weight (0-24 hours) and Day 2 was the weight taken at 24 hrs. Day 3 is 48 to 72 hours. Newborns in the study seem to experience weight losses comparable to reports in the literature [8,9]. The percentage of weight lost peaked at 60 hours (i.e., 3rd day) with a mean 6.57% loss (SD 2.51, range 1.83 to 13.06, n = 96).

In a systematic review of early weight loss patterns, 11 studies demonstrated a mean loss of about 6% with a standard deviation of about 2 (median was also about 6%); the nadir (point of lowest weight) was the third day [8]. Martens and Romphf determined exclusively breastfed babies lost a mean of 5.49% and supplemented breastfed babies lost an average of 5.52% in hospital [9]. With weight measures only in hospital, the nadir of weight loss may not have been reached [9]. MacDonald et al. completed a prospective study and found breastfed babies lost a median of 6.6% of birth weight (95 centile = 11.8%) within a median time of 2.7 days [40]. Crossland et al. developed a centile chart (n = 111) capturing weight loss in the first two weeks postpartum [41]. They also showed that breastfed neonates average a loss of 6.4% of birth weight with the majority reaching the point of maximum weight loss on the third day [41].

### Strengths and limitations

Strengths include: (a) data collected prospectively; we collected data about the three key variables which were not available in the medical records; (b) participants used the same scale to ensure internal consistency; (c) weight measurements every 12 hours for the first 72 hours permitted detection of the nadir of weight loss; and (d) measurements post discharge added valuable information.

The limitations are attributable to data collection issues. Although our main concern was fluid shifts, we could not collect voids separate from stools. The late decision to collect additional data about fluids in the final two hours before birth meant a small sample for this analysis. The first weeks following birth are an intense time for parents, and data were frequently missed. We did not attempt to input missing data regarding weights and fluids, because we could not be certain of the direction (e.g., should weight go up, down, or stay the same). Babies managed to void and stool when their diapers were off. Parents were asked to document missed output, and we estimated to account for the loss.

### Reconsider birth weight as baseline

Clinicians debate the limits of acceptable neonatal weight loss in the first days. Current clinical practice guidelines recommend interventions, including extra assessments or supplementation with formula, when weight loss exceeds 7% [4-7]. Some authors identify a loss of ≥10% as a sign of breastfeeding inadequacy [35,37]. Weight loss, in this case, is the percentage of weight lost from the first weight measured (i.e., birth weight). Birth weight as a baseline against which to assess weight loss is a universal choice, but it lacks sufficient empirical evidence.

It appears that the neonates in our study experienced diuresis in the first 24 hours as evidenced by the positive correlation of the first 24-hour output to both the maternal two-hour prebirth IV fluids and the weight loss at 24 hours. With birth weight as baseline, newborns may have an artificially high reference point for weight loss. Resetting baseline to a point after the diuresis has occurred (i.e., the newborn's weight has stabilized) would be a better gauge for assessment. This premise is supported by van Dommelen et al. who determined a 10% rule of thumb produces false positives (i.e., not a good indicator to detect hypernatremic dehydration) [42].

We ran frequency analyses of percentage weight loss to contrast two possible baselines (see Additional file 1). With a 24-hour baseline, 2.3% lost between 7 and 10%, and none lost in excess of 10%. In contrast, one third of the newborns lost between 7 and 10% of their birth weight and 7.3% lost more than 10%. With a 24-hour baseline, 90% regained their baseline weight by Day 9 (n = 88). Whereas, 64% regained birth weight by Day 9 (n = 97). By Day 14, 12% had not regained their birth weight (n = 102), but 99% had regained their 24 hour weight measurement (n = 93).

Intuitively, clinicians and parents want to see the neonate return to birth weight. If it is an inflated measurement, then the expectations for a return to birth weight in the first days are questionable. In the dialysis literature, the term "dry weight" is used to describe a patient's weight without additional fluid, and this measurement is the patient's post-dialysis goal weight [43]. The neonates in this study appear to reach their dry weight around 24 hours, although the timing of this iatrogenic weight loss might depend on birth practices.

### Future research

Further research is needed to understand the effects of iatrogenic factors such as maternal fluids during parturition. Evidence is needed to confirm why and how timing of maternal fluid is a factor; especially IV fluids administered in the last hours before birth. Tracking maternal output might provide insight into the phenomena. Researchers should note the strong correlation between maternal IV fluids and epidurals and birth types, as these latter two factors may be not be the source of weight loss.

A study with diaper weights grouped by 12 hours might determine the peak of diuresis. It is possible that diuresis continues to 36 hours, but we could not analyze output and weight loss at 36 hours because the diapers were weighed in 24 hour segments.

The findings about delayed lactogenesis II should be investigated further. The positive correlation between maternal fluids and onset of lactogenesis II was a serendipitous finding. The finding that delayed lactogenesis II was related to newborn weight loss was not unexpected, but that the effect was not evident in the regression model at 60 hours, only showing up at 72 hours, was unanticipated. In the literature, the frequency of delayed onset of lactogenesis II ranges widely from 22% to 44% [35,44]. The modifiable factors that affect onset of lactogenesis II and the effects of delayed onset need to be better understood.

Research is needed to correlate morbidity and mortality to newborn weight loss. Using a percentage (i.e., 10%) as a red flag does not appear to have a connection to morbidity. The criterion seems reversed. For example, Manganaro et al. divided their sample based on 10% then completed blood tests in the > 10% loss group, instead of determining the relationship between morbidity and percentage of weight loss [37].

Researchers who plan studies about neonatal weight loss need to be careful to use hours and not days for their protocols. For example, diapers for Day 1 can be interpreted many ways (e.g., participants could restart the count the following morning). Stipulating from birth to 12 or 24 hours is clearer. Additionally, conditions for daily weight measurements could be specified for consistency (e.g., weigh before feeds).

## Conclusions

The phenomenon of newborn weight loss is complex. The prevailing attitude seems to be weight loss must be prevented and controlled. With this research, we found evidence that maternal IV fluids during parturition are related to neonatal output and newborn weight loss; specifically, a correction in fluid balance not requiring intervention. The effect seems time limited, and further weight loss after the first 72 hours is not likely connected to maternal fluids and should not be dismissed as a fluid correction.

We believe weight change data is a valuable assessment tool, as newborn weight loss can be a sign of lack of feeding or underlying morbidity. At the same time, we emphasize weight measurements should only be a tool for assessment and not the basis for clinical decisions. A complete evaluation is needed, and observations of neonatal behaviour, frequency and amounts of output, and feeding behaviours should also contribute to breastfeeding assessments.

## Competing interests

The authors declare that they have no competing interests.

## Authors' contributions

JN-W completed the original study for her doctoral dissertation. AKW supervised the dissertation. WEP, WG, and DLG were committee members. JN-W conceived the study and all authors contributed to the study design and to interpretation of the results. JN-W wrote the first draft of this manuscript and the co-authors contributed to the pre-submission revisions. All authors approved the final manuscript.

## Authors' information

JN-W RN IBCLC PhD is an experienced nurse and lactation consultant who has worked with mothers and their babies in hospital and community settings. JN-W is an assistant professor at the University of Ottawa, and the focus of her research program is breastfeeding and human lactation.

AKW RN MSc PhD is dean of the Trent-Fleming School of Nursing. AKW specializes in women's cardiovascular health and quantitative methods.

WEP RN PhD is an assistant professor at the School of Nursing at the University of Ottawa, Ontario, Canada. WP is an experienced perinatal nurse, and she is building a research program in perinatal health.

WG BSc PhD is a physiologist, researcher, and professor at the University of Ottawa. WG's research interests include preterm labour and the role of multidrug resistance in fetal development.

DLG RN PhD is an epidemiologist in the psychiatry department at Queen's University. DLG specializes in quantitative methods and data analysis.

## Supplementary Material

Additional file 1 **Frequency of percentage weight loss with two different baselines (N = 109)**. Click here for file
