# Supplementary material for: An observational study of associations among maternal fluids during parturition, neonatal output, and breastfed newborn weight loss
Source: Int Breastfeed J. 2011 Aug 15;6:9. doi: 10.1186/1746-4358-6-9 (PMC3174114; doi:10.1186/1746-4358-6-9)
Supplement: Additional file 1 — Frequency of percentage weight loss with two different baselines (N = 109). [file 1746-4358-6-9-S1.PDF]

Additional file

Frequency of percentage weight loss with two different baselines (N = 109)

	Newborn Weight Loss from Birth				Newborn Weight Loss from 24 hours			
Timing	*Sample Size*	*< 7%*	*7 to 10 %*	*> 10%*	*Sample Size*	*< 7%*	*7 to 10 %*	*> 10%*
12 hrs	95	100%	Ø	Ø	N/A			
24 hrs	97	94.8%	5.2%	Ø				
36 hrs	98	75.5%	24.5%	Ø	89	100%	Ø	Ø
48 hrs	105	59.0%	40.0%	1.0%	96	100%	Ø	Ø
**60 hrs**	**96**	**59.4%**	**33.3%**	**7.3%**	**87**	**97.7%**	**2.3%**	**Ø**
72 hrs	100	67.0%	29.0%	4.0%	91	98.9%	1.1%	Ø
Day 4	103	80.6%	15.5%	3.9%	92	100%	Ø	Ø
Day 5	96	87.5%	11.5%	1.0%	87	100%	Ø	Ø
Day 6	96	92.7%	6.3%	1.0%	86	100%	Ø	Ø
Day 7	101	93.1%	5.9%	1.0%	91	100%	Ø	Ø
